# Perfluorinated compounds in adults and their association with fasting glucose and incident diabetes: a prospective cohort study

**DOI:** 10.1186/s12940-022-00915-2

**Published:** 2022-10-26

**Authors:** Seung Min Chung, Dong-Gyu Heo, Ju-Hyun Kim, Ji Sung Yoon, Hyoung Woo Lee, Jong-Yeon Kim, Jun Sung Moon, Kyu Chang Won

**Affiliations:** 1 Division of Endocrinology and Metabolism, Department of Internal Medicine, Yeungnam University College of Medicine, Hyunchung-Ro 170, Nam-Gu, Daegu, 42415 Republic of Korea; 2 College of Pharmacy, Yeungnam University, Gyeongsan, 38541 Korea; 3 Department of Physiology, Yeungnam University College of Medicine, Daegu, Republic of Korea

**Keywords:** Fasting glucose, Incident diabetes, Perfluorooctanoic acid, Perfluorooctanesulfonic acid

## Abstract

**Background:**

The endocrine disruption of perfluorinated compounds is an emerging issue. We aimed to examine the association of serum perfluorooctanoic acid (PFOA) and perfluorooctanesulfonic acid (PFOS) levels with incident diabetes and fasting serum glucose concentration.

**Methods:**

This prospective cohort study was based on an urban-based cohort subpopulation from the Korean Genome and Epidemiology Study. Serum samples (600 µL) were received from 100 participants in the normoglycemic baseline survey (2004–2013), and concentrations of PFOA and PFOS were measured using mass spectrometry. The incidence of diabetes was tracked in the follow-up survey (2012–2016).

**Results:**

The mean age was 56.4 years (men, 59%). The median serum PFOA and PFOS concentrations were 4.29 ng/mL and 9.44 ng/mL, respectively. PFOA and PFOS concentrations differed according to age, sex, and residential area. After 60 months, 23 patients had diabetes. Log-transformed PFOA (lnPFOA) and log-transformed PFOS (lnPFOS) were significantly higher in those who transitioned to diabetes than in those who did not (both *p* < 0.05). After multivariate adjustment, lnPFOA (coefficient = 6.98, 95% CI -0.04–14*, p* = 0.054) and lnPFOS (coefficient = 7.06, 95% CI -0.96–15.08*, p* = 0.088) predicted increased fasting glucose without statistical significance. In addition, lnPFOA, but not lnPFOS, significantly predicted incident diabetes (HR = 3.98*,* 95% CI 1.42–11.1, *p* < 0.01).

**Conclusion:**

Exposure to PFOA and PFOS may have a potential dysglycemic effect. In particular, exposure to PFOA increased the risk of diabetes. Further research with larger sample size is warranted.

**Supplementary Information:**

The online version contains supplementary material available at 10.1186/s12940-022-00915-2.

## Background

Perfluorinated compounds are organic pollutants distributed in air, water, and soil that are resistant to environmental degradation processes and eventually bioaccumulate in humans [1]. The two representative perfluorinated compounds are perfluorooctanoic acid (PFOA, C_8_HF_15_O_2_) and perfluorooctanesulfonic acid (PFOS, C_8_HF_17_O_3_S).

In the 1990s, a toxic chemical PFOA was leaked from the DuPont company in the United States (US) without permission, consequently, cancer incidence and birth defects in the residential area rapidly increased [2]. Accordingly, the US conducted a large-population study to identify the relationship between perfluorinated compounds and human diseases. Exposure to perfluorinated compounds is consistently reported to be associated with hypercholesterolemia [3, 4]. Although debate continues, perfluorinated compounds are associated with glucose metabolism [5, 6], thyroid dysfunction [7], immunity, semen quality, and reproduction [4, 8].

Emerging studies are investigating the impact of exposure to perfluorinated compounds on glucose homeostasis. Of these, several studies have demonstrated positive association between serum perfluorinated compounds and decreased insulin secretion [9], increased insulin resistance [10], fasting glucose [11], and 2-h glucose levels [12]. However, some studies showed non-significant or inverse associations between perfluorinated compounds and glucose homeostasis markers [13–15]. These conflicting results might be due to differences in ethnic-, regional-, gender-, and age-specific exposure; therefore, further prospective studies are required.

In Korea in June 2018, perfluorinated compounds were over-detected in tap water in Daegu [16], suggesting the need for studies to determine the effects of perfluorinated compounds on human health. However, the relevant studies in the Korean population are scarce. We aimed to analyze the serum concentrations of PFOA and PFOS and explore their relationship with increased fasting glucose and incident diabetes in Korean adults.

## Methods

This prospective cohort study was based on an urban-based cohort from the Korea Biobank Project and Korean Genome and Epidemiology Study, which was conducted for men and women aged 40–69 who visited the nationwide health screening centers [17]. The study included participants from the baseline survey (2004–2013) whose serum samples and clinical data were available and who subsequently participated in the follow-up survey (2012–2016). Participants who had diabetes at the baseline survey were excluded. The clinical data and serum samples (600 µL/person, stored frozen at − 80 °C) were obtained from 100 participants who met the eligibility criteria. This study was approved by the Institutional Review Board of Yeungnam University Medical Center in Daegu, South Korea (IRB no. YUMC2019-08–064), and the need for informed consent was waived.

### Measurements of PFOA and PFOS concentration

Serum PFOA and PFOS were measured by chromatography-mass spectrometry (LC–MS/MS) using an Agilent 6460 triple quadrupole mass spectrometer coupled with 1260 high-performance liquid chromatography (Agilent Technologies, Santa Clara, CA, USA). The lack of PFOA-free and PFOS-free human serum remained a major challenge in previous studies; thus, previous studies involved the use of surrogate matrices, such as calf serum, bovine serum albumin, and artificial cerebrospinal fluids [18, 19]. To overcome this limitation, we developed the methodology using pooled normal human serum (Innovative Research, Inc. Novi, MI, USA) and ^13^C-labeled PFOA and PFOS (Wellington Laboratories, Guelph, Ontario, Canada). The ^13^C_8_PFOA and ^13^C_8_PFOS were used as calibrates for the quantification. The ^13^C_4_PFOA and ^13^C_4_PFOS were used as internal standards for ensuring that the analytical results of samples were obtained consistently. This method was validated based on the US Food and Drug Administration’s guidelines for bioanalytical method validation [20]. Details of the analysis are published elsewhere [21].

After validation, the method was applied to evaluate the levels of exposure to PFOA and PFOS in 100 Korean serum samples. Each assay required 160 µL of human serum. The lower limit of quantification was 0.05 ng/mL, and the assay response was linear at 10 ng/mL for both PFOA and PFOS. Serum PFOA and PFOS concentrations exceeded the quantification range and were reanalyzed after tenfold dilution.

### Covariates

The participants’ information was collected: age (40–49; 50–59; 60–69), sex (male; female), region (Gangwon; Gyeonggi/Incheon; Gyeongsang; Busan/Daegu), and new onset comorbidities of diabetes, hypertension, dyslipidemia, myocardial infarction, and cerebrovascular accident. Education level was classified into less than middle school, high school graduate, and college graduation or higher. Income status was classified into < 1.5, 1.5–3, 3–6, and > 6 million KRW/mo. Smoking and drinking status was classified into never-, ex-, and current- smoker (or drinker). Data on whether undergoing regular moderate-intensity exercise was collected.

Height, weight, waist circumference, and blood pressure (BP) were measured by trained staff members. Body mass index (BMI) was calculated as body weight in kilograms divided by height in meters squared (kg/m^2^). Venous blood samples were collected after an 8-h fast. The levels of glycated hemoglobin (HbA1c), fasting glucose, creatinine, total cholesterol, high-density lipoprotein (HDL) cholesterol, low-density-lipoprotein (LDL) cholesterol, and triglycerides were measured.

### Outcomes

All participants were normoglycemic in the baseline survey, and incident diabetes was tracked in the follow-up survey, where participants were classified as participants with no diabetes (PND), with prediabetes (PPD), and with diabetes (PD). Prediabetes was diagnosed if fasting glucose was 100–126 mg/dL or HbA1c was 5.7–6.5%. Diabetes was diagnosed if fasting glucose was ≥ 126 mg/dL and HbA1c was ≥ 6.5% or if an endocrinologist diagnosed diabetes after the baseline survey [22]. The incident diabetes was identified based on the date described in the follow-up survey questionnaire, or the date of follow-up examination for those who were first diagnosed at follow-up survey.

### Statistical analysis

Statistical analyses were performed using R version 3.6.3 package and GraphPad Prism 9.0 software (GraphPad Software Inc., San Diego, CA, USA). Serum PFOA and PFOS concentrations were presented as median with interquartile ranges (IQR) or geometric means ± geometric standard deviations. Other variables were expressed as means ± standard deviations and as numbers and percentages for categorical variables.

Differences between groups were assessed using independent sample *t*-tests or one-way analysis of variance (Tukey's multiple comparisons) for continuous variables and chi-squared tests for categorical variables. Linear and Cox regression analyses were used to assess the effects of PFOA and PFOS on fasting glucose and incident diabetes. Multivariate regression models were established by adjusting the demographics, socioeconomic factors, and metabolic parameters. Coefficients or hazard ratios (HRs) were reported with 95% confidence intervals. Statistical significance was set at *p* values < 0.05.

## Results

### Baseline characteristics

The characteristics of 100 participants at baseline survey are shown in Table 1. The mean age was 56.4 ± 8.3 and the male-to-female ratio was 1.4:1. Of total participants, 20%, 14%, 36%, and 30% lived in Gangwon, Gyeonggi/Incheon, Gyeongsang, and Busan/Daegu, respectively. Education level and income status were predominantly distributed in high school graduates and 3–6 million KRW/mo. Current smokers and drinkers accounted for 22% and 46%, respectively. Approximately 61% of participants were on regular exercise.

**Table 1:** Baseline characteristics of 100 participants

Age	56.4 ± 8.3 [range: 40, 69]
Male:Female	59:41
Region, n	
Gangwon	20
Gyeonggi/Incheon	14
Gyeongsang	36
Busan/Daegu	30
Education level, n	
less than middle school	25
High school graduate	42
College graduation or higher	33
Income, KRW/mo, n	
< 1.5 million	25
1.5–3 million	27
3–6 million	32
> 6 million	11
Smoke, n	
Never	59
Ex-smoker (> 20pk)	19
Current-smoker (> 20pk)	22
Drink, n	
Never	50
Ex-drinker	4
Current-drinker	46
Regular moderate-intensity exercise, n	
No	39
Yes	61

### Serum concentrations of PFOA and PFOS

**Fig. 1:**
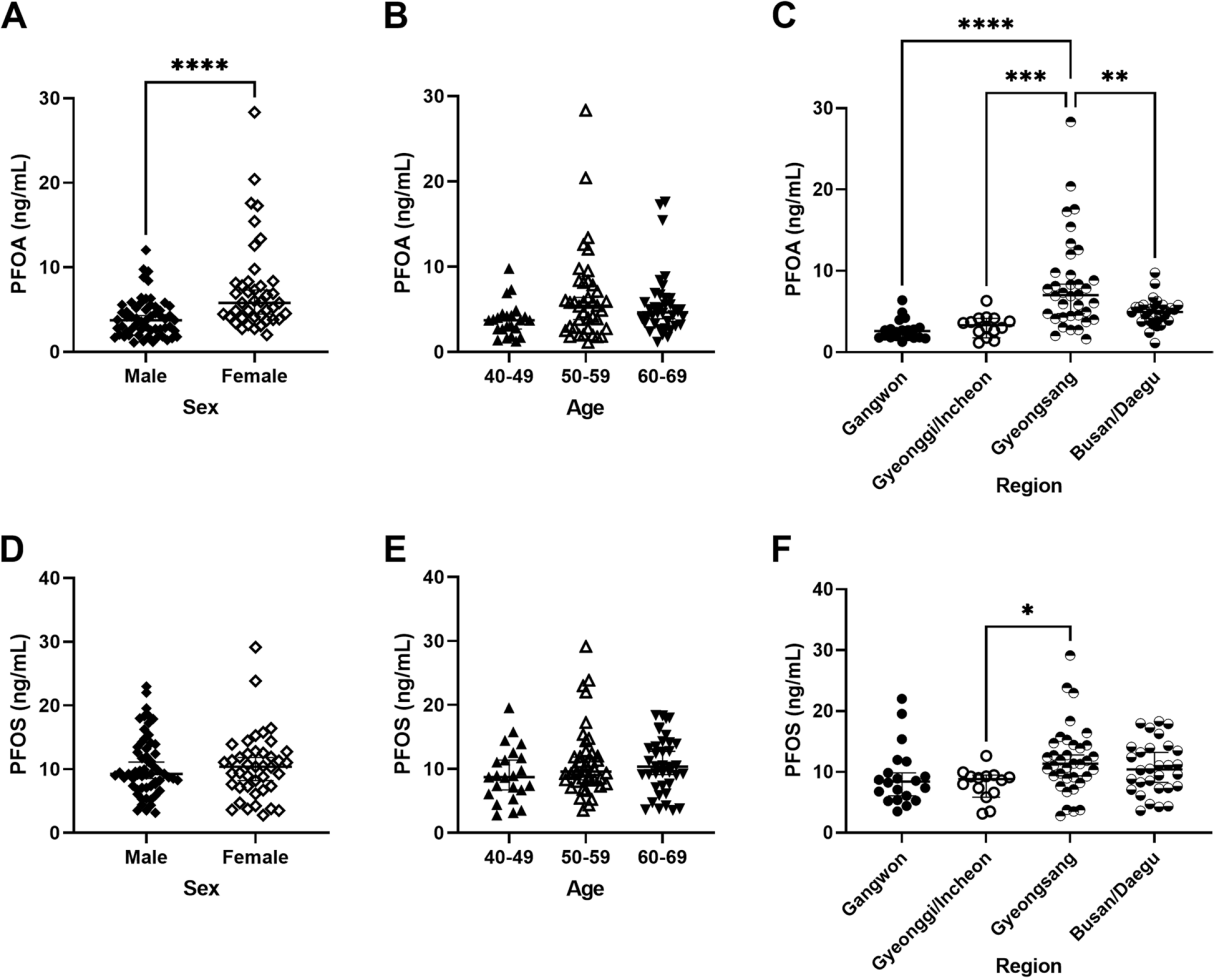
Concentration of PFOA according to **A** sex, **B** age, and **C** residential area, and concentration of PFOS according to **D** sex, **E** age, and **F** residential area. *p < 0.05, **p < 0.01, ***p < 0.001, ****p < 0.0001. The bar presents geometric mean and geometric standard deviation (SD)

The serum concentrations of PFOA and PFOS ranged from 0.42 to 28.34 ng/mL and from 0.81 to 57.55 ng/mL, respectively. The median serum PFOA and PFOS concentrations were 4.29 ng/mL (interquartile range [IQR]: 2.80–6.31) and 9.44 ng/mL (IQR: 7.30–12.78), respectively. Serum PFOA and PFOS concentrations differed according to sex, age, and residential area (Fig. 1). Particularly, the concentration of PFOA was higher in females than in males (7.5 ng/mL vs. 4.0 ng/mL; *p* < 0.0001) and was significantly higher in participants living in the Gyeongsang region (8.3 ng/mL) than in those living in Gangwon (2.7 ng/mL), Gyeonggi/Incheon (3.3 ng/mL), and Busan/Daegu (4.9 ng/mL; *p* < 0.01 for all comparison). The PFOS concentration was significantly higher in participants living in the Gyeongsang region (11.9 ng/mL) than in those living in Gyeonggi/Incheon (3.3 ng/mL; *p* < 0.05). Participants with PFOA concentration higher than median were less educated and had lower income than those with PFOA concentration lower than median (both *p* < 0.01); and participants with PFOS higher than median tended to have very low or high income and ratio of never- and ex- smokers higher than those with PFOS lower than median (both *p* < 0.05; Supplementary Table 1).

### Relationship between PFOA, PFOS, and baseline metabolic parameters

**Fig. 2:**
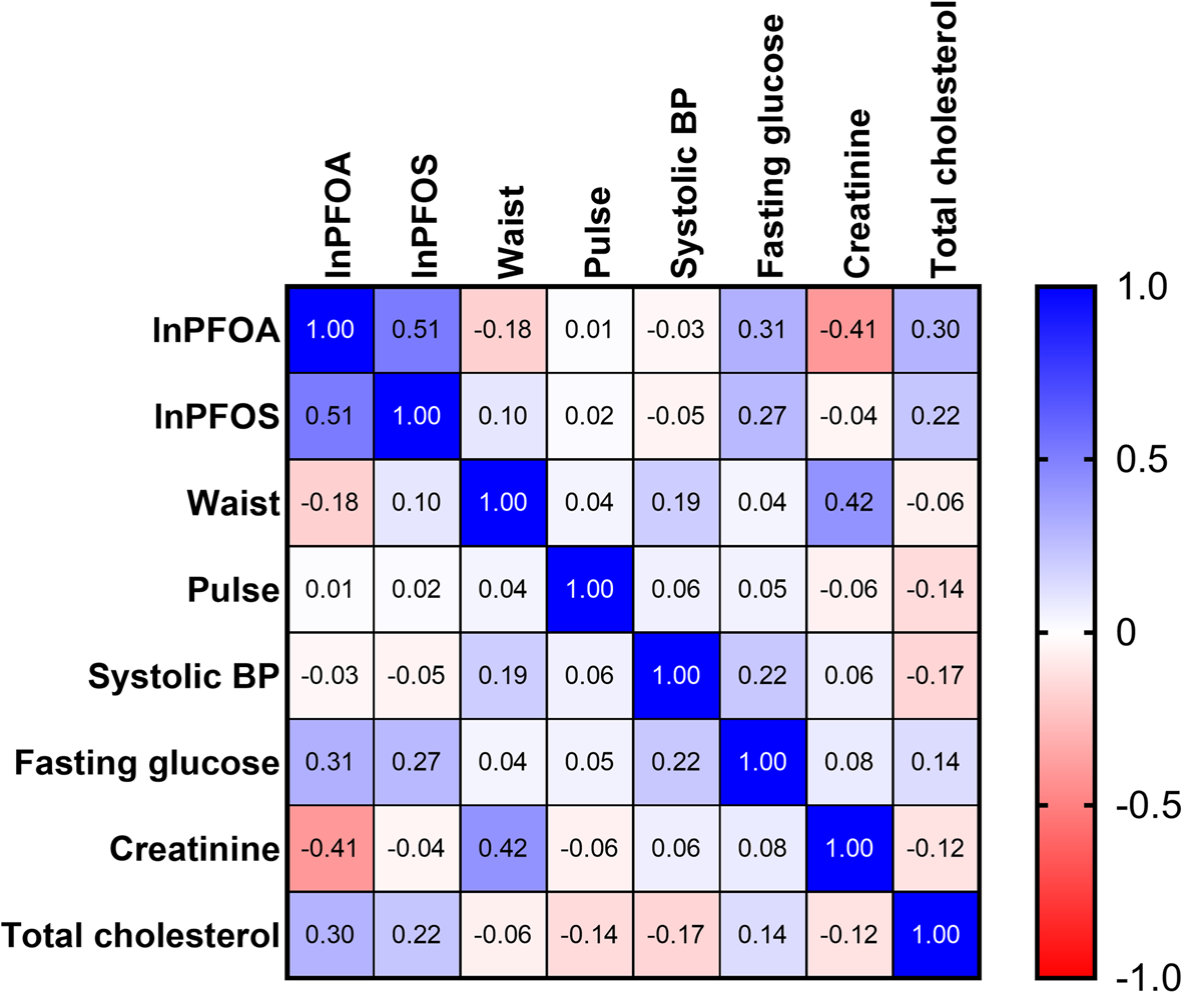
Correlation of lnPFOA, lnPFOS, and baseline metabolic parameters among participants without diabetes at baseline (*n* = 100). Correlation between variables is presented as Pearson correlation r. The blue color box has a positive correlation, and a red color box has a negative correlation

PFOA and PFOS were log-transformed due to their skewed distribution (log-transformed PFOA [lnPFOA]: median 1.23 ng/mL, IQR 0.78–1.66 ng/mL; log-transformed PFOS [lnPFOS]: median 2.17 ng/mL, IQR 1.85–2.52 ng/mL). The relationship among lnPFOA, lnPFOS, and baseline metabolic parameters (waist circumference, pulse rate, systolic BP, fasting glucose, creatinine, and total cholesterol) was analyzed using correlation analysis and presented as a heatmap (Fig. 2). lnPFOA and lnPFOS were highly correlated to each other (*r* = 0.51, *p* < 0.0001). Both lnPFOA and lnPFOS showed highly positive correlation with fasting glucose (*r* = 0.31 and 0.27, both *p* < 0.01) and total cholesterol (*r* = 0.30 and 0.22, *p* < 0.01). Additionally, lnPFOA was negatively associated with creatinine (*r* = -0.41, *p* < 0.0001).

### Follow-up characteristics according to the occurrence of diabetes

After 60.3 ± 21.0 months of follow-up, 30 and 23 participants had transitioned to prediabetes and diabetes, respectively. Participant characteristics according to the occurrence of diabetes are described in more detail in Table 2. Compared to the PND and PPD groups, participants in the PD group were significantly older in age (68.1 ± 7.6 years at follow-up) and had male dominance (87%) (both *p* < 0.01). The proportion of new-onset hypertension, dyslipidemia, cerebrovascular accident, and myocardial infarction did not differ among the PND, PPD, and PD groups. Proportions of education level less than middle school (43.5%) and income status less than 1.5 million KRW/mo (43.5%) were prominent in the PD group compared with those the PND and PPD group; however, these differences were not statistically significant. Approximately 70% of participants were never smokers in the PND and PPD group and 73% were ex- or current smokers in the PD group (*p* < 0.01). In the PD group, the pulse rate was faster and the metabolic profiles (fasting glucose, HbA1c, and HDL cholesterol) were poorer (*p* < 0.05).

**Table 2:** Characteristics according to the occurrence of diabetes

	**Non-diabetes (*n* = 47)**	**Prediabetes (*n* = 30)**	**Diabetes (*n* = 23)**	***P***
Age (years)	59.1 ± 9.5	60.1 ± 7.3	68.1 ± 7.6	< 0.001
Men, n (%)	26 (55.3)	13 (43.3)	20 (87.0)	< 0.01
New-onset comorbidities, n (%)				
Hypertension	2 (4.3)	3 (10.0)	1 (4.3)	0.577
Dyslipidemia	6 (12.8)	2 (6.7)	5 (21.7)	0.286
Cerebrovascular accident	0 (0.0)	1 (3.3)	1 (4.3)	0.281
Myocardial infarction	1 (2.1)	0 (0.0)	0 (0.0)	1
Education level, n (%)				
Less than middle school	9 (19.1)	6 (20.0)	10 (43.5)	0.173
High school graduate	20 (42.6)	13 (43.3)	9 (39.1)	
College graduation or higher	18 (38.3)	11 (36.7)	4 (17.4)	
Income (KRW/mo), n (%)				
< 1.5 million	10 (23.3)	5 (17.2)	10 (43.5)	0.053
1.5–3 million	9 (20.9)	9 (31.0)	9 (39.1)	
3–6 million	18 (41.9)	10 (34.5)	4 (17.4)	
> 6 million	6 (14.0)	5 (17.2)	0 (0.0)	
Smoke, n (%)				
Never	33 (70.2)	20 (66.7)	6 (26.1)	< 0.01
Ex-smoker (> 20 pk)	6 (12.8)	3 (10.0)	10 (43.5)	
Current smoker (> 20 pk)	8 (17.0)	7 (23.3)	7 (30.4)	
Drink, n (%)				
Never	23 (48.9)	17 (56.7)	10 (43.5)	0.369
Ex-drinker	3 (6.4)	1 (3.3)	0 (0.0)	
Current drinker	21 (44.7)	12 (40.0)	13 (56.5)	
Regular moderate-intensity exercise, n (%)				
No	19 (40.4)	15 (50.0)	10 (43.5)	0.71
Yes	28 (59.6)	15 (50.0)	13 (56.5)	
Waist circumference (cm)	83.0 ± 8.3	81.8 ± 6.8	86.4 ± 8.1	0.098
BMI (kg/m^2^)	24.2 ± 2.3	24.1 ± 2.5	24.6 ± 2.7	0.739
Pulse (/min)	70.1 ± 9.4	73.6 ± 10.9	77.4 ± 9.2	< 0.05
Systolic BP (mmHg)	124.3 ± 12.3	123.1 ± 12.7	129.7 ± 19.5	0.375
Diastolic BP (mmHg)	74.6 ± 9.2	72.9 ± 9.3	76.5 ± 14.1	0.534
Fasting glucose (mg/dL)	89.2 ± 5.9	99.8 ± 7.5	125.9 ± 24.7	< 0.001
HbA1c (%)	5.3 ± 0.3	5.8 ± 0.3	6.6 ± 0.6	< 0.001
Creatinine (mg/dL)	0.9 ± 0.3	0.8 ± 0.2	1.0 ± 0.3	0.067
Total cholesterol (mg/dL)	194.0 ± 37.9	205.8 ± 37.9	179.1 ± 30.9	< 0.05
HDL cholesterol (mg/dL)	52.7 ± 11.7	55.2 ± 14.5	45.3 ± 9.4	< 0.01
LDL cholesterol (mg/dL)	117.7 ± 36.6	122.2 ± 33.9	103.1 ± 27.0	0.065
Triglyceride (mg/dL)	126.4 ± 108.9	141.8 ± 57.4	151.3 ± 74.9	0.534

### Differences in PFOA and PFOS concentrations according to the occurrence of diabetes

**Fig. 3:**
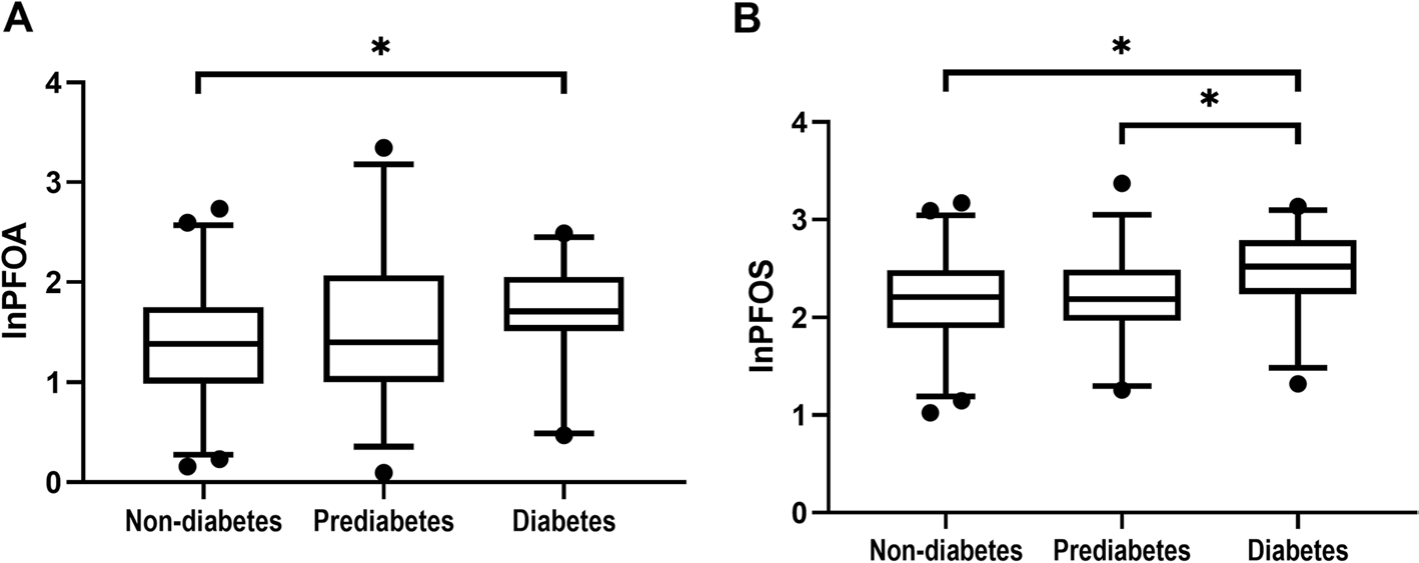
Concentrations of **A** lnPFOA and **B** lnPFOS according to the diabetes status. *p < 0.05. The box indicates median and IQR. The whiskers indicate 5–95 percentiles. lnPFOA, log-transformed perfluorooctanoic acid; lnPFOS, log-transformed perfluorooctanesulfonic acid; IQR, interquartile range

The median concentrations of baseline lnPFOA in the PND, PPD, and PD groups were 1.38 ng/mL (IQR: 0.99–1.76 ng/mL), 1.40 ng/mL (IQR: 1.01–2.07 ng/mL), and 1.71 ng/mL (IQR: 1.51–2.05 ng/mL), respectively. The concentration of baseline lnPFOA was significantly higher in the PD group than in the PND group (*p* < 0.05; Fig. 3A). The median concentrations of baseline lnPFOS in the PND, PPD, and PD groups were 2.21 ng/mL (IQR: 1.89–2.48 ng/mL), 2.19 ng/mL (IQR: 1.97–2.49 ng/mL), and 2.52 ng/mL (IQR: 2.24–2.79 ng/mL), respectively. The concentration of baseline lnPFOS was significantly higher in the PD group than in the PND and PPD groups (both *p* < 0.05; Fig. 3B).

### Impact of PFOA and PFOS on fasting glucose and incident diabetes

The impact of lnPFOA and lnPFOS on fasting glucose and incident diabetes was analyzed after adjusting for age, sex, income, smoking status, waist circumference, systolic blood pressure, and total cholesterol levels at baseline (Table 3). After 5 years, a higher lnPFOA (coefficient = 6.98, 95% CI -0.04–14, *p* = 0.054) and lnPFOS (coefficient = 7.06, 95% CI -0.96, 15.08, *p* = 0.088) predicted increased fasting glucose without statistical significance. The predictive value for incident diabetes was significant with higher lnPFOA (HR = 3.98, 95% CI 1.42–11.1, *p* = 0.008), but not with higher lnPFOS (HR = 1.71, 95% CI 0.37, 7.96, *p* = 0.488).

**Table 3:** Impact of lnPFOA and lnPFOS on fasting glucose and incident diabetes

**Follow-up survey (2012–2016)**	**lnPFOA**		**lnPFOS**	
	Coefficient (95% CI)	*P*	Coefficient (95% CI)	*P*
Fasting glucose, mg/dL ^a^	6.98 (-0.04, 14)	0.054	7.06 (-0.96, 15.08)	0.088
	HR (95% CI)	*P*	HR (95% CI)	*P*
Incident diabetes ^b^	3.98 (1.42, 11.1)	0.008	1.71 (0.37, 7.96)	0.488

Age, sex, income, smoking status, waist circumference, systolic BP, and total cholesterol levels at baseline were considered as covariates. The variables were eliminated through a backward stepwise regression

*Abbreviations: CI* Confidence interval, *HR* Hazard ratio, *lnPFOA* log-transformed perfluorooctanoic acid, *lnPFOS* log-transformed perfluorooctanesulfonic acid

^a^Linear regression analysis

^b^Cox regression analysis

## Discussion

This study was performed using human bioresources and the clinical cohort data of 100 Korean participants received from the Korea Biobank Project and the Korean Genome and Epidemiology Study. The serum PFOA and PFOS concentrations differed according to age, sex, and residential area. The lnPFOA and lnPFOS had a positive correlation with fasting glucose levels in participants without diabetes at baseline. Higher baseline concentrations of lnPFOA and lnPFOS was likely linked to increase levels of fasting glucose at the 5-year follow-up. The predictive value of higher lnPFOA for incident diabetes at the 5-year follow-up was statistically significant; however, this was not the case for higher lnPFOS concentrations.

The Korean participants in this study had similar or lower concentrations of PFOA and PFOS (median: 4.29 ng/mL and 9.44 ng/mL, respectively) than the US nationwide survey participants (mean PFOA and PFOS, 4.13 ng/mL and 13.2 ng/mL, respectively) [23]. Although serum concentrations of PFOA and PFOS appear to have a decreasing trend in the US and Norway [24], more than half of the general population still have PFOA and PFOS concentrations higher than the acceptable guideline values of Germany (PFOA < 2 ng/mL and PFOS < 5 ng/mL) [25].

The primary sources of PFOA and PFOS exposure are mainly food and indoor dust, accounting for up to 50% of cases in women [26]. In addition, perfluorinated compounds are water-soluble; hence, they are found in groundwater, surface water contaminated with industrial wastewater, and water not affected by point sources [27]. Study participants exposed to water contaminated with perfluorinated compounds released from industrial plants reportedly have mean PFOA and PFOS serum concentrations of 32.9 ng/mL and 19.2 ng/mL, respectively [2]. In Korea, the Gyeongsang region has the highest PFOA and PFOS concentrations in soil and water samples [28], consistent with the relatively high serum concentrations found in the present study; hence, larger studies on industrial pollution in the Korean population are warranted.

The mechanism of perfluorinated compounds induced toxicity is still unclear; however, the pathways closely related to human health are p53/mitochondrial pathway, PI3K-AKT, and tumor necrosis factor-α/nuclear factor-κB [29, 30]. Perfluorinated compounds bind to nuclear receptors such as aryl hydrocarbon receptor, constitutive androstane receptor, pregnane X receptor, peroxisome proliferator-activated receptor, and farnesoid X receptor, which increase organ inflammation and oxidative stress, mitochondrial dysfunction, resulting in insulin resistance, metabolic syndrome, and liver disease [31, 32].

Studies of perfluorinated compounds and their association with diabetes have been reported. The exposure to PFOA or PFOS increased the risk of diabetes 1.5–2.6 times in US participants [33, 34]. Among Chinese participants, exposure to PFOA or PFOS was associated with glucose homeostasis and risk of diabetes, which was stronger in women [35]. In contrast, a few studies have reported that PFOA or PFOS have neutral effects on glucose homeostasis [36, 37]. It is necessary to investigate the concentrations of PFOA and PFOS in various ethnicities and to investigate the appropriate cut-off that causes endocrine disturbance.

The strengths of this study are as follows: this is the first study to explore the effects of perfluorinated compounds on diabetes in Korean adults, with a diverse city distribution of participants. Additionally, a sensitive LC–MS/MS method was used to analyze PFOA and PFOS concentrations in human serum. Finally, the hazardous effect of PFOA on dysglycemia was revealed, which provides insight into a field of growing interest.

Despite these strengths, this study has some limitations. First, it is not generalizable to the entire Korean population because of the small number of study participants. Second, dysglycemia was not severe in those who transitioned to diabetes at follow-up; the mean HbA1c was 6.6%, and the mean fasting glucose level was 126 mg/dL. Finally, since fasting insulin levels were not measured, it was impossible to evaluate insulin sensitivity and resistance.

## Conclusions

In conclusion, the serum concentrations of PFOA and PFOS vary according to age, sex, and region. PFOA and PFOS may have the potential to increase fasting glucose level; PFOA especially affects the incidence of diabetes. Special attention to the adverse effects of high PFOA on glucose dysregulation is required for exposed individuals.

## Supplementary Information

**Additional file 1: Supplementary Table 1.** Association between PFOA, PFOS and covariates.

## Data Availability

Biospecimens and data were obtained from the Korean Genome Analysis Project (4845–301), the Korean Genome and Epidemiology Study (4845–302), and the Korea Biobank Project (4851–307, KBP-2014–000) that were supported by the Korea Centers for Disease Control & Prevention, Republic of Korea (NBK-19111102–01-01). The datasets generated and analyzed during the current study are not publicly available because they were obtained from the third party but are available from the corresponding author on reasonable request.

## Abbreviations

lnPFOA: Log-transformed perfluorooctanoic acid;
lnPFOS: Log-transformed perfluorooctanesulfonic acid
PFOA: Perfluorooctanoic acid
PFOS: Perfluorooctanesulfonic acid
PND: Participants with no diabetes
PPD: Participants with prediabetes
PD: Participants with diabetes

## References

[1] Jensen AA, Leffers H (2008). Emerging endocrine disrupters: perfluoroalkylated substances. Int J Androl.

[2] Frisbee SJ, Brooks AP, Maher A, Flensborg P, Arnold S, Fletcher T, Steenland K, Shankar A, Knox SS, Pollard C (2009). The C8 health project: design, methods, and participants. Environ Health Perspect.

[3] Eriksen KT, Raaschou-Nielsen O, McLaughlin JK, Lipworth L, Tjonneland A, Overvad K, Sorensen M (2013). Association between plasma PFOA and PFOS levels and total cholesterol in a middle-aged Danish population. PLoS ONE.

[4] Steenland K, Fletcher T, Savitz DA (2010). Epidemiologic evidence on the health effects of perfluorooctanoic acid (PFOA). Environ Health Perspect.

[5] Roth K, Petriello MC (2022). Exposure to per- and polyfluoroalkyl substances (PFAS) and type 2 diabetes risk. Front Endocrinol (Lausanne).

[6] Qi W, Clark JM, Timme-Laragy AR, Park Y (2020). Per- and Polyfluoroalkyl substances and obesity, type 2 diabetes and non-alcoholic fatty liver disease: a review of epidemiologic findings. Toxicol Environ Chem.

[7] Kim HY, Kim KN, Shin CH, Lim YH, Kim JI, Kim BN, Hong YC, Lee YA. The relationship between Perfluoroalkyl Substances concentrations and thyroid function in early childhood: a prospective cohort study. Thyroid 2020; 30(11):1556–65. 10.1089/thy.2019.0436 32368952

[8] Vested A, Ramlau-Hansen CH, Olsen SF, Bonde JP, Kristensen SL, Halldorsson TI, Becher G, Haug LS, Ernst EH, Toft G (2013). Associations of in utero exposure to perfluorinated alkyl acids with human semen quality and reproductive hormones in adult men. Environ Health Perspect.

[9] Domazet SL, Grontved A, Timmermann AG, Nielsen F, Jensen TK (2016). Longitudinal associations of exposure to Perfluoroalkylated substances in childhood and adolescence and indicators of adiposity and glucose metabolism 6 and 12 years later: the European Youth Heart Study. Diabetes Care.

[10] Kim JH, Park HY, Jeon JD, Kho Y, Kim SK, Park MS, Hong YC (2016). The modifying effect of vitamin C on the association between perfluorinated compounds and insulin resistance in the Korean elderly: a double-blind, randomized, placebo-controlled crossover trial. Eur J Nutr.

[11] Duan Y, Sun H, Yao Y, Meng Y, Li Y (2020). Distribution of novel and legacy per-/polyfluoroalkyl substances in serum and its associations with two glycemic biomarkers among Chinese adult men and women with normal blood glucose levels. Environ Int.

[12] Alderete TL, Jin R, Walker DI, Valvi D, Chen Z, Jones DP, Peng C, Gilliland FD, Berhane K, Conti DV (2019). Perfluoroalkyl substances, metabolomic profiling, and alterations in glucose homeostasis among overweight and obese Hispanic children: a proof-of-concept analysis. Environ Int.

[13] Fisher M, Arbuckle TE, Wade M, Haines DA (2013). Do perfluoroalkyl substances affect metabolic function and plasma lipids?–Analysis of the 2007–2009, Canadian Health Measures Survey (CHMS) Cycle 1. Environ Res.

[14] Fleisch AF, Rifas-Shiman SL, Mora AM, Calafat AM, Ye X, Luttmann-Gibson H, Gillman MW, Oken E, Sagiv SK (2017). Early-life exposure to Perfluoroalkyl substances and childhood metabolic function. Environ Health Perspect.

[15] Nelson JW, Hatch EE, Webster TF (2010). Exposure to polyfluoroalkyl chemicals and cholesterol, body weight, and insulin resistance in the general U.S. population. Environ Health Perspect.

[16] Panic after chemicals found in Daegu, South Korea drinking water 2018. https://pfasproject.com/2018/06/29/panic-after-chemicals-found-in-daegu-south-korea-drinking-water/.

[17] National biobank of Korea. http://www.nih.go.kr/biobank/EgovPageLink.do?link=main%2Fcontent%2FNationalHRBank%2FEngPop.

[18] Kuklenyik Z, Needham LL, Calafat AM (2005). Measurement of 18 perfluorinated organic acids and amides in human serum using on-line solid-phase extraction. Anal Chem.

[19] Kato K, Basden BJ, Needham LL, Calafat AM (2011). Improved selectivity for the analysis of maternal serum and cord serum for polyfluoroalkyl chemicals. J Chromatogr A.

[20] FDA (2018). Bioanalytical method validation.

[21] Heo DG, Lee DC, Kwon YM, Seol MJ, Moon JS, Chung SM, Kim JH (2022). Simultaneous determination of perfluorooctanoic acid and perfluorooctanesulfonic acid in Korean sera using LC-MS/MS. J Chromatogr B Analyt Technol Biomed Life Sci.

[22] Kim MK, Ko SH, Kim BY, Kang ES, Noh J, Kim SK, Park SO, Hur KY, Chon S, Moon MK (2019). 2019 clinical practice guidelines for type 2 diabetes mellitus in Korea. Diabetes Metab J.

[23] Kato K, Wong LY, Jia LT, Kuklenyik Z, Calafat AM (2011). Trends in exposure to polyfluoroalkyl chemicals in the U.S. population: 1999–2008. Environ Sci Technol.

[24] Vestergren R, Cousins IT (2009). Tracking the pathways of human exposure to perfluorocarboxylates. Environ Sci Technol.

[25] Apel P, Angerer J, Wilhelm M, Kolossa-Gehring M (2017). New HBM values for emerging substances, inventory of reference and HBM values in force, and working principles of the German human biomonitoring commission. Int J Hyg Environ Health.

[26] Haug LS, Huber S, Becher G, Thomsen C (2011). Characterisation of human exposure pathways to perfluorinated compounds–comparing exposure estimates with biomarkers of exposure. Environ Int.

[27] Post GB, Cohn PD, Cooper KR (2012). Perfluorooctanoic acid (PFOA), an emerging drinking water contaminant: a critical review of recent literature. Environ Res.

[28] Choi G-H, Lee D-Y, Jeong D-K, Kuppusamy S, Lee YB, Park B-J, Kim J-H (2017). Perfluorooctanoic acid (PFOA) and perfluorooctanesulfonic acid (PFOS) concentrations in the South Korean agricultural environment: a national survey. J Integr Agric.

[29] Du G, Sun J, Zhang Y (2018). Perfluorooctanoic acid impaired glucose homeostasis through affecting adipose AKT pathway. Cytotechnology.

[30] Li K, Gao P, Xiang P, Zhang X, Cui X, Ma LQ (2017). Molecular mechanisms of PFOA-induced toxicity in animals and humans: Implications for health risks. Environ Int.

[31] Wahlang B (2018). Exposure to persistent organic pollutants: impact on women's health. Rev Environ Health.

[32] Takacs ML, Abbott BD (2007). Activation of mouse and human peroxisome proliferator-activated receptors (alpha, beta/delta, gamma) by perfluorooctanoic acid and perfluorooctane sulfonate. Toxicol Sci.

[33] He X, Liu Y, Xu B, Gu L, Tang W (2018). PFOA is associated with diabetes and metabolic alteration in US men: national Health and Nutrition Examination survey 2003–2012. Sci Total Environ.

[34] Sun Q, Zong G, Valvi D, Nielsen F, Coull B, Grandjean P (2018). Plasma concentrations of perfluoroalkyl substances and risk of Type 2 diabetes: a prospective investigation among US Women. Environ Health Perspect.

[35] Zeeshan M, Zhang YT, Yu S, Huang WZ, Zhou Y, Vinothkumar R, Chu C, Li QQ, Wu QZ, Ye WL (2021). Exposure to isomers of per- and polyfluoroalkyl substances increases the risk of diabetes and impairs glucose-homeostasis in Chinese adults: Isomers of C8 health project. Chemosphere.

[36] Cardenas A, Gold DR, Hauser R, Kleinman KP, Hivert MF, Calafat AM, Ye X, Webster TF, Horton ES, Oken E (2017). Plasma concentrations of per- and polyfluoroalkyl substances at baseline and associations with glycemic indicators and diabetes incidence among high-risk adults in the diabetes prevention program trial. Environ Health Perspect.

[37] Preston EV, Rifas-Shiman SL, Hivert MF, Zota AR, Sagiv SK, Calafat AM, Oken E, James-Todd T. Associations of per- and polyfluoroalkyl substances (PFAS) with glucose tolerance during pregnancy in project viva. J Clin Endocrinol Metab. 2020;105(8):e2864–76. 10.1210/clinem/dgaa328 PMC7320827 32480407

