# Supplementary material for: Perfluorinated compounds in adults and their association with fasting glucose and incident diabetes: a prospective cohort study
Source: Environ Health. 2022 Oct 26;21:101. doi: 10.1186/s12940-022-00915-2 (PMC9597959; doi:10.1186/s12940-022-00915-2)
Supplement: Supplementary file 1 — Additional file 1: Supplementary Table 1. Association between PFOA, PFOS and covariates. [file 12940_2022_915_MOESM1_ESM.docx]

**Supplementary Table 1. Association between PFOA, PFOS and covariates**

|  | PFOA <4.29 ng/mL  (n=50) | PFOA ≥4.29 ng/mL  (n=50) | p | PFOS <9.44ng/mL (n=50) | PFOS ≥9.44 ng/mL (n=50) | p |
| --- | --- | --- | --- | --- | --- | --- |
| Education level, n (%) |  |  |  |  |  |  |
| less than middle school | 9 (18.0) | 16 (32.0) | <0.001 | 12 (24.0) | 13 (26.0) | 0.134 |
| High school graduate | 13 (26.0) | 29 (58.0) |  | 17 (34.0) | 25 (50.0) |  |
| College graduation or higher | 28 (56.0) | 5 (10.0) |  | 21 (42.0) | 12 (24.0) |  |
| Income, KRW/mo, n(%) |  |  |  |  |  |  |
| <1.5 million | 8 (16.7) | 17 (36.2) | 0.008 | 8 (17.0) | 17 (35.4) | 0.036 |
| 1.5-3 million | 10 (20.8) | 17 (36.2) |  | 19 (40.4) | 8 (16.7) |  |
| 3-6 million | 22 (45.8) | 10 (21.3) |  | 16 (34.0) | 16 (33.3) |  |
| >6 million | 8 (16.7) | 3 (6.4) |  | 4 (8.5) | 7 (14.6) |  |
| Smoke, n(%) |  |  |  |  |  |  |
| Never | 28 (56.0) | 31 (62.0) | 0.112 | 28 (56.0) | 31 (62.0) | 0.026 |
| Ex-smoker (>20pk) | 7 (14.0) | 12 (24.0) |  | 6 (12.0) | 13 (26.0) |  |
| Current-smoker (>20pk) | 15(30.0) | 7 (14.0) |  | 16 (32.0) | 6 (12.0) |  |
| Drink, n(%) |  |  |  |  |  |  |
| Never | 25 (50.0) | 25 (50.0) | 0.165 | 26 (52.0) | 24 (48.0) | 0.511 |
| Ex-drinker | 4 (8.0) | 0 (0.0) |  | 3 (6.0) | 1 (2.0) |  |
| Current-drinker | 21 (42.0) | 25 (50.0) |  | 21 (42.0) | 25 (50.0) |  |
| Regular moderate-intensity exercise, n(%) |  |  |  |  |  |  |
| No | 21 (42.0) | 18 (36.0) | 0.682 | 24 (48.0) | 15 (30.0) | 0.101 |
| Yes | 29 (58.0) | 32 (64.0) |  | 26 (52.0) | 35 (70.0) |  |
